# Prediction of Surface Roughness of an Abrasive Water Jet Cut Using an Artificial Neural Network

**DOI:** 10.3390/ma14113108

**Published:** 2021-06-05

**Authors:** Mirko Ficko, Derzija Begic-Hajdarevic, Maida Cohodar Husic, Lucijano Berus, Ahmet Cekic, Simon Klancnik

**Affiliations:** 1 Faculty of Mechanical Engineering, University of Maribor, Smetanova ulica 17, 2000 Maribor, Slovenia; lucijano.berus@um.si (L.B.); simon.klancnik@um.si (S.K.); 2 Faculty of Mechanical Engineering, University of Sarajevo, Vilsonovo setaliste 9, 71000 Sarajevo, Bosnia and Herzegovina; begic@mef.unsa.ba (D.B.-H.); cohodar@mef.unsa.ba (M.C.H.); cekic@mef.unsa.ba (A.C.)

**Keywords:** abrasive water jet cutting, surface roughness, stainless steel, artificial neural network

## Abstract

The study’s primary purpose was to explore the abrasive water jet (AWJ) cut machinability of stainless steel X5CrNi18-10 (1.4301). The study analyzed the effects of such process parameters as the traverse speed (TS), the depth of cut (DC), and the abrasive mass flow rate (AR) on the surface roughness (*Ra*) concerning the thickness of the workpiece. Three different thicknesses were cut under different conditions; the *Ra* was measured at the top, in the middle, and the bottom of the cut. Experimental results were used in the developed feed-forward artificial neural network (ANN) to predict the *Ra*. The ANN’s model was validated using k-fold cross-validation. A lowest test root mean squared error (RMSE) of 0.2084 was achieved. The results of the predicted *Ra* by the ANN model and the results of the experimental data were compared. Additionally, as TS and DC were recognized, analysis of variance at a 95% confidence level was used to determine the most significant factors. Consequently, the ANN input parameters were modified, resulting in improved prediction; results show that the proposed model could be a useful tool for optimizing AWJ cut process parameters for predicting *Ra*. Its main advantage is the reduced time needed for experimentation.

## 1. Introduction

Abrasive water jet (AWJ) machining is one of the beneficial machining processes used to cut various engineering materials. The importance of this kind of machining lies in the fact that it has a small heat-affected zone, no thermal distortion, a small machining force, high flexibility, and a good surface finish. Overall, the AWJ machining processes achieve high precision and accuracy of complex parts. Process parameters such as the water pressure, the abrasive type, the abrasive mass flow rate (AR), and the traverse speed (TS) influence the cutting surface quality and the overall process efficiency. The prediction of surface roughness depending on process parameters with their optimization represents a challenging task and attracts researchers worldwide.

Overview articles [1,2] provide a comprehensive review of the AWJ process parameters. Some researchers even try to control the surface texture with process parameters [3]. Many traditional modeling techniques, such as regression analysis, do not provide satisfactory results, especially when the relationship between the target function and the influencing parameters is non-linear, as is usually the case in complex phenomena (such as AWJ machining); this indicates the appropriateness of artificial neural network (ANN) based methods for overall cutting process modeling [4]. For modeling and optimization of any machining processes, ANNs [5], an adaptive neuro-fuzzy inference system (ANFIS) [6,7], and other intelligent techniques [8] are common. ANN and the regression model were used for surface roughness prediction in the AWJ cutting of AA 7075 aluminum alloy [9]. Additionally, different approaches have been applied for the investigation of surface roughness in the AWJ process, such as fuzzy logic in [10], the Taguchi-based analysis of variance method in [11,12,13], the multi-objective genetic algorithm (MOGA) in [14], and the regression method in [10,15]. Liu et al. [16] developed quadratic regression models to predict the penetration depth and surface roughness in abrasive water jet turning of alumina ceramics using a response surface methodology with a Box–Behnken design. Different kinds of materials were subjected to the AWJ process, among them, carbon steel S235 [14], Hardox steel [15], magnesium alloy [10], aluminum alloy [17,18], titanium alloy [19], marble [20], aluminum/magnesium hybrid metal matrix composites [11], a lanthanum phosphate/yttria composite [12], and Nimonic C236 superalloy [21].

An overview of the current research shows that this topic has been gaining importance in recent years. Most researchers are trying to control and optimize the process parameters for various materials and with various methods [22]. From the presented literature overview, different approaches were used to investigate the AWJ cutting surface roughness [23]. Most researchers are trying to control and optimize the process parameters for various materials and with various methods [22]. From the presented literature overview, different approaches were used to investigate the AWJ cutting surface roughness [23,24]. Because of the many process variables and complex physical phenomena taking place during the AWJ cutting, machine learning methods for modeling the process are appropriate. Ganovska B. et al. [25] used the ANN for on-line control of the AWJ process. Hence, in this study, the surface roughness was predicted in the AWJ cutting of stainless steel using the ANN method at different depths of cut. The presented research deals just with the average surface roughness in the direction of a water jet. It distinguishes between the top, middle, and bottom sections of the AWJ cut at various material depths. The cutting conditions change along with the depth; therefore, it was essential to predict the surface roughness at the entire depth of cut, which is very important for cutting the materials of different thicknesses and is even more critical for the larger materials’ thicknesses. Therefore, this study aimed to predict the surface roughness using the ANNs method during abrasive water jet cutting of stainless steel of different thicknesses. Then, by using ANOVA, we determined which process parameters affected the surface roughness significantly concerning the thickness of the workpiece.

This article is structured as follows. The introduction section presents the overview of the research of the AWJ cutting surface roughness modeling. The second section describes the experimental test setup and presents the experimental results; it also includes modeling of the cutting surface roughness with ANN, together with method validation and performance metrics. The third section presents the results and factor analysis for statistical assessment of the influence of variables on the cutting surface roughness. The last section concludes with important remarks and directs further research and implementation of the developed model.

## 2. Materials and Methods

### 2.1. Experimental Setup Description

The experiments were conducted using a Flow Mach 4 abrasive water jet machine (Flow Waterjet, Kent WA, USA). The material of the machined workpiece used in the experiments was X5CrNi18-10 (1.4301) stainless steel plates of three different thicknesses, 5 mm, 10 mm, and 15 mm. The material was austenitic stainless steel, which is difficult to machine from a traditional cutting process standpoint and is often processed with an AWJ [23]. After evaluating the process parameters, we selected those with the most significant influence on the cut quality for the design of experiments (DOE). The constant process parameters and their values are presented in Table 1.

The most influential (DOE input variables) process parameters are the nozzle stand-off distance, the traverse speed, and the abrasive mass flow rate [26]. The latter two input process parameters were selected for analysis in the present study, since the stand-off distance, already after the preliminary test, did not show a considerable influence. Table 2 shows the traverse speed and the abrasive mass flow rate values used in the experiments.

**Figure 1:**
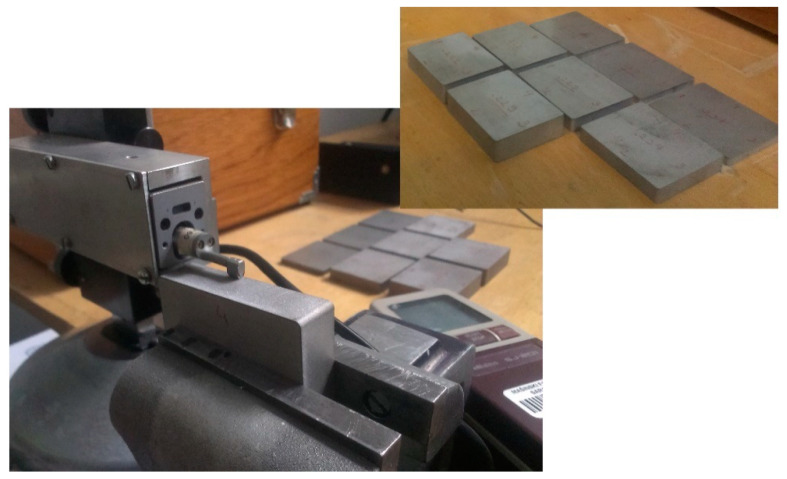
The sample to measure surface roughness with the measuring equipment.

The surface roughness with a cut-off of 0.8 mm (on the cut surface) was measured with the Mitutoyo SJ-201 Surftest (Mitutoyo, Kawasaki, Japan), as shown in Figure 1.

**Figure 2:**
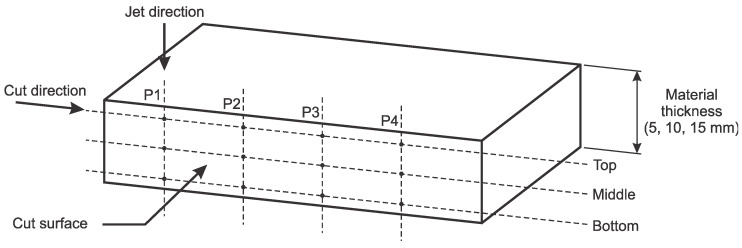
Schematic view of the cut surface with sections and locations where surface roughness was measured.

The *Ra* was used to evaluate the surface roughness; it is one of the most-studied AWJ process parameters in research and industry. The surface roughness was measured at three different sections in the jet direction (top, middle, and bottom), as shown in Figure 2. The roughness was measured at four locations (P1, P2, P3, and P4) along the length of the cut surface, as shown in Figure 2.

### 2.2. Experimental Results

Altogether, 108 data points, 36 sets of 3 experiments, were conducted in this investigation. The *Ra* values of each experimental trial are listed in Table 3, Table 4 and Table 5.

### 2.3. Artificial Neural Networks

ANNs simulate the human brain; they have been used to model various problems in the economic, social, medical, and engineering sciences. ANNs are data-driven self-adaptive methods capable of arbitrary adjustment to model the system without any explicit specification of functional form for the underlying model, and, consequently, they can map any function with arbitrary accuracy [27]. 

An ANN consists of an input layer of nodes, one or more hidden layers, and an output layer. The input layer in our case consisted of neurons that represent different thicknesses, AR, TS, and DC (independent variables). The hidden layer is a collection of neurons that provide an intermediate connection between the input and output layers. The hidden layer of the neural network maps the inputs into image space Г. The number of neurons in the output layer determines the number of dependent variables. The output value of the i-th neuron $y_{i}$ was determined using Equation (1):

(1) $$y_{i}=ʄ\left(\varepsilon_{i}\right)=ʄ\left(\sum^{\;}\omega_{ij}x_{j}-\vartheta_{i}\right)$$

where, $ʄ\left(\varepsilon_{i}\right)$ denotes a transfer function such as sigmoid or logarithmic, $\varepsilon_{i}$ the potential of the i-th neuron, and $x_{j}$ the j-th input vector value transmitted through a neuron. In our case, the output was presented as an average of P1, P2, P3, and P4 values (mean Ra). The weight matrix and threshold vector coefficients were denoted as $\omega_{ij}$ and $\vartheta_{i},$ respectively; both were adopted iteratively using a specific training procedure, with the intent to minimize the sum of squared differences E in Equation (2). The $y_{o}$ and $t_{o}$ vectors represent the ANN’s output value and the actual/desired output value (based on the experiment); the overall summation runs over all output neurons o.

(2) $$E=\sum_{o}1/2\;{\left(y_{o}-t_{o}\right)}^{2}$$

when using a multilayer feed-forward ANN, the network’s architecture is one of the most important factors [28]. Since an over-simplified (shallow with fewer neurons) ANN is less flexible [29], complex ANNs are prone to over-fitting [30] and are computationally expensive. There is no simple formula for determining the number of neurons and hidden layers; the ANN topology depends critically on the number of training cases, the amount of noise, and the overall complexity of the given problem [31,32]. Smaller ANN network architectures are faster (fewer neurons/layers involved in calculations), easier to build (and maintain), and offer better generalization ability.

### 2.4. Pre-Processing Validation and Performance Metrics 

Normalization mapping of the input variable values on (−1, 1) was performed as a preprocessing step. The special k-fold cross-validation procedure with k=36 was adopted for evaluating the performance of the proposed ANNs. This means that the data was divided into 36 subsets; 35 datapoints subsets were used for training and the remaining one was used for testing the ANNs’ performance. Each fold contained all representative positions throughout the depth of the cut, belonging to the same cut. Datapoints belonging to a specific fold have the same thickness, AR, and TS values (and different positions throughout the depth of the cut). For better generalization, the whole 36-fold procedure was repeated 10 times (wherein fold members are left unaltered). Performance metrics include the mean average error (MAE) and the root mean squared error (RMSE).

## 3. Results and Discussion

**Figure 3:**
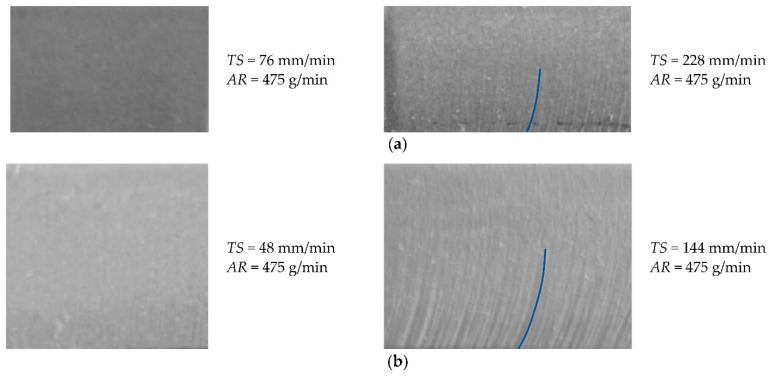
The images of cut surface: (**a**) for the material thickness of 10 mm and (**b**) for the material thickness of 15 mm.

Figure 3 shows the images of the cut surfaces, which were cut at the maximum and minimum traverse speed and an abrasive mass flow rate of 475 g/min for material thicknesses of 10 mm and 15 mm. At a minimum traverse speed for both material thicknesses (76 mm/min for a 10 mm and 48 mm/min for a 15 mm), no grooves were visible at the cut surface. However, at a maximum traverse speed (228 m/min for 10 mm and 144 m/min for 15 mm), the grooves were easily visible, especially on the lower half of the material’s thickness. Since the cut surface images do not offer quantitative information, the research focused on measuring the surface roughness at three different sections in the jet direction (top, middle, and bottom) and discussing only those values.

### 3.1. ANN Results

Table 6 shows the initially tested feed-forward ANN configurations. The presented ANNs were comprised of two hidden layers (four in the first and eight neurons in the second hidden layer). Different training procedures and different transfer functions were adopted. The different training procedures included scaled conjugate gradient (“trainscg”), gradient descent with adaptive learning rate (“traingda”), Levenberg–Marquardt (“trainlm”), and Bayesian regularization (“trainsbr”) backpropagations. The adopted transfer functions were logarithmic (“logsig”) and linear (“purelin”). The best results (testMAE=0.2046, testRMSE=0.2397) were achieved with the scaled conjugate gradient learning method and the logarithmic transfer function; the stated configuration was adopted further on.

**Figure 4:**
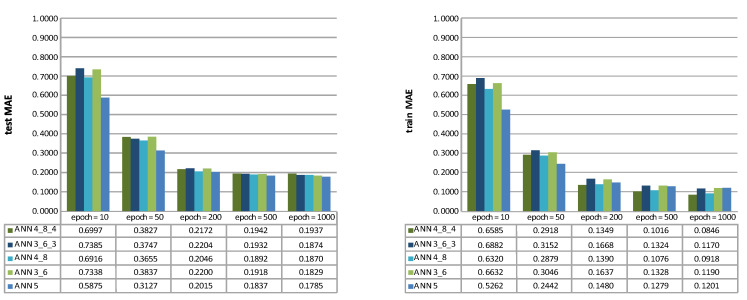
Test (**left**) and train MAE (**right**) of five different ANN topologies; ANN 5, ANN 3_5, ANN 4_8, ANN 3_6_3, and ANN 4_8_4.

Feed-forward ANNs with five different topologies ANN 5, ANN 3_5, ANN 4_8, ANN 3_6_3, and ANN 4_8_4 were used, comprising one to three hidden layers. For clarification, the ANN 5 topology contained one hidden layer with five neurons. The ANN 3_6_3 contained three hidden layers with three neurons in the first hidden layer, six in the second, and three in the third hidden layer. As stated, all the neurons in the hidden layers adopted the logarithmic (“logsig”) transfer function and the scaled conjugate gradient learning method (“trainscg”)-based learning method. For the ANNs’ validation, the k-fold cross-validation was used with 36 folds, wherein each fold contained three data points with different depth sections (bottom, middle, and upper). The learning procedure lasted for five different epoch durations (10, 50, 200, 500, and 1000 iterations). After that, the ANN’s performance was assessed based on the MAE (Figure 4) and the RMSE (Figure 4) metrics.

**Figure 5:**
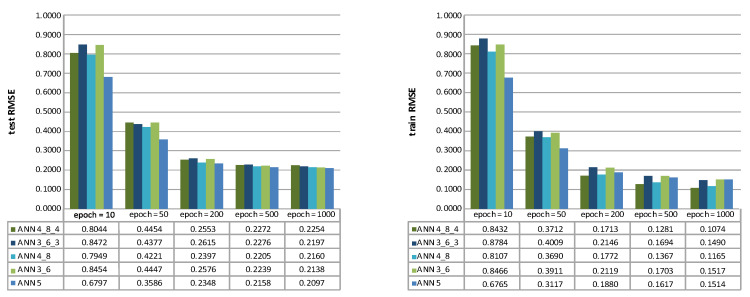
Test (**left**) and train RMSE (**right**) of five different ANN topologies; ANN 5, ANN 3_5, ANN 4_8, ANN 3_6_3, and ANN 4_8_4.

The best test set results were achieved with ANN 5 (testMAE=0.1785, testRMSE=0.2097) after 1000 epochs. The graphs (Figure 4 and Figure 5) indicate that a higher number of hidden layers in ANNs, yield to be have proven less efficient in surface roughness (mean *Ra*) prediction, mainly when a shorter learning duration was adopted (the epoch number was less than 200).

**Figure 6:**
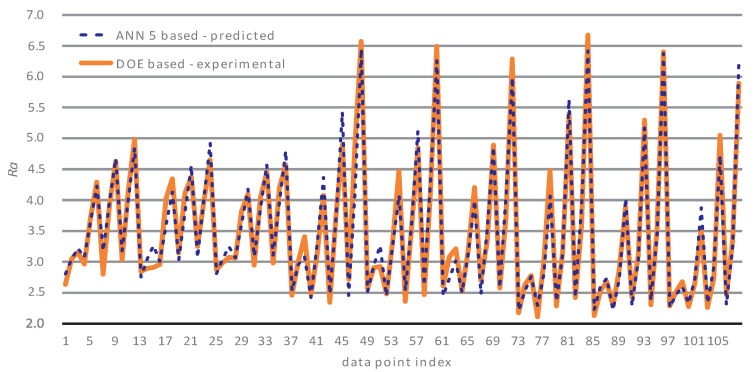
Experimental surface roughness vs. predicted surface roughness of the training data.

Figure 6 presents a comparison plot of the experimental and predicted surface roughness values of the training data. Let us stress that the predicted surface roughness values were averaged over all 10 iterations of the ANN’s validation test set. The average percentage error between the predicted surface roughness (based on the ANN 5 configuration trained for 1000 epochs—discussed in the paragraph above) and the experimental values was 4.4973%.

### 3.2. Analysis of Variance

A statistical method, analysis of variance, was applied to identify which process parameters affected the surface roughness significantly concerning the thickness of the workpiece. The degree of freedom (DF), the squares (SS), the square means (MS), the *F*-values, and the *p*-values were presented for each factor (process parameter). In this study, the *p*-value was taken at a level of 0.05, and the results were validated for a confidence level of 95%. If the value of *p* was less than 0.05, the factor was statistically significant. If the *p*-value was greater than 0.05, the factor was not statistically significant at a 95% confidence level. The percentage of contribution (PC) was used to analyse the significance of the process parameters. 

**Figure 7:**
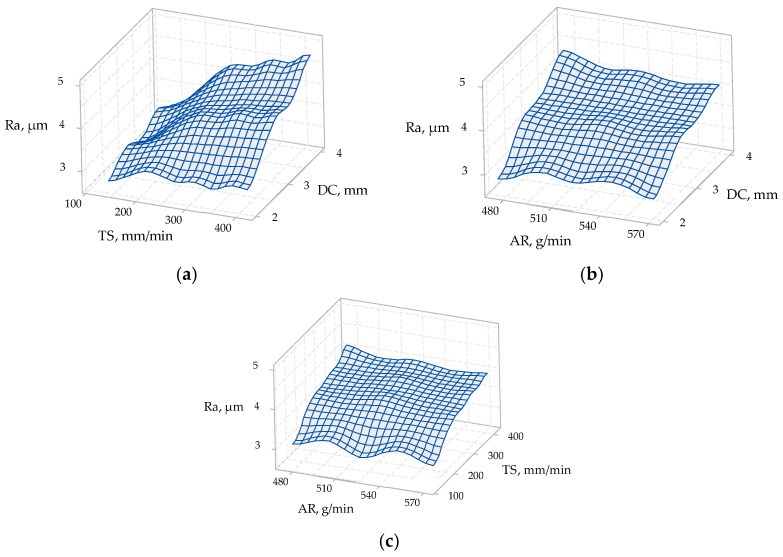
3D surface plots of process parameters vs. surface roughness of 5 mm material thickness: (**a**) traverse speed (TS) vs. depth of cut (DC) on surface roughness (*Ra*); (**b**) abrasive mass flow rate (AR) vs. depth of cut (DC) on surface roughness (*Ra*); (**c**) abrasive mass flow rate (AR) vs. traverse speed (TS) on surface roughness (*Ra*).

The ANOVA results for the materials of 5 mm thickness are presented in Table 7, and Figure 7 presents the 3D surface plots of process parameters vs. surface roughness for 5 mm material thickness.

From Table 7, the *p*-values were 0.966 for AR, 0.0 for TS, and 0.0 for DC. The TS and the DC were shown to influence the surface roughness, unlike the AR. The strongest influence can be attributed (based on ANOVA) to the DC.

It can be observed in Figure 7a,b that the depth of cut was the most impactful factor affecting the surface roughness. Additionally, it can be seen in Figure 7a,c that, with the increase in the traverse speed, the surface roughness increased, except at a 2 mm depth of cut (top section). Moreover, it can be observed in Figure 7b,c that the abrasive mass flow rate did not have a significant effect on the surface roughness; although, in Figure 7b, the surface roughness decreased slightly with the increase in the abrasive mass flow rate at the 4 mm depth of cut (bottom section).

**Figure 8:**
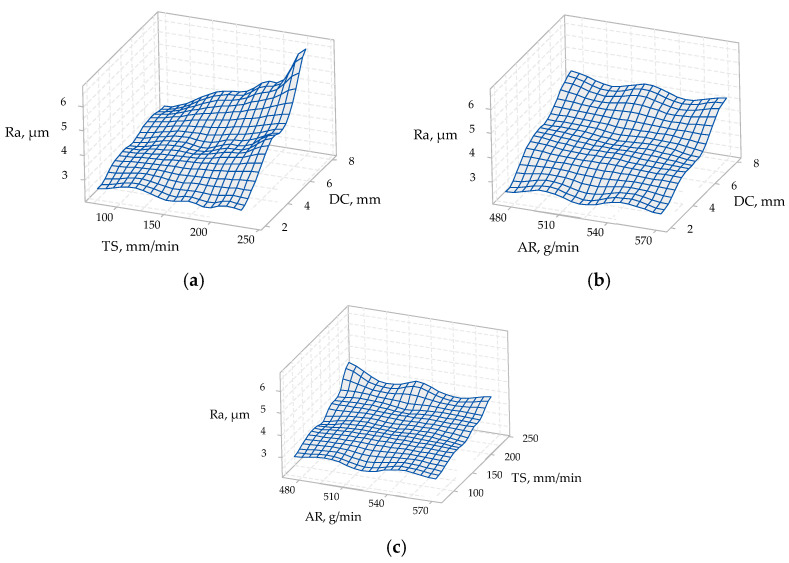
3D surface plots of process parameters vs. surface roughness of 10 mm material thickness: (**a**) traverse speed (TS) vs. depth of cut (DC) on surface roughness (*Ra*); (**b**) abrasive mass flow rate (AR) vs. depth of cut (DC) on surface roughness (*Ra*); (**c**) abrasive mass flow rate (AR) vs. traverse speed (*i*) on surface roughness (*Ra*).

The ANOVA results for materials of 10 mm thickness are presented in Table 8. Figure 8 presents the 3D surface plots of process parameters and their influence on the surface roughness value for materials of 10 mm thickness.

From Table 8, the *p*-value for AR was 0.866, and it was 0.0 for TS and for DC. Based on ANOVA (with the adopted 95% confidence levels), the highest percentage contribution was obtained for the depth of cut, setting it as the factor with the most influence on the surface roughness.

It can be seen in Figure 8a,b that the surface roughness increased with the increase in the depth of the cut. A significant increase in surface roughness was observed in Figure 8a at the maximum value of the traverse speed, especially at an 8 mm depth of cut (bottom section). Figure 8c shows that the surface roughness decreased slightly with the increase in the abrasive mass flow rate at the maximum value of the traverse speed.

ANOVA results for materials of 15 mm thickness are presented in Table 9. Figure 8 depicts the 3D surface plots of the process parameters and their influence on the surface roughness value for materials of 15 mm thickness.

From Table 9, the *p*-value for AR was 0.639, and it was 0.0 for TS and for DC. It can be noticed that the highest percentage contribution, based on ANOVA, was obtained for the depth of cut, recognizing it as the most influential factor on the surface roughness, shown in Table 7 and Table 8. Based on the ANOVA results (shown in Table 7, Table 8 and Table 9), the highest percentage contribution for the abrasive mass flow rate was obtained for the 15 mm material thickness. Thus, it can be concluded that, with the increase in the material thickness, the impact of the AR parameter on the surface roughness also increased.

**Figure 9:**
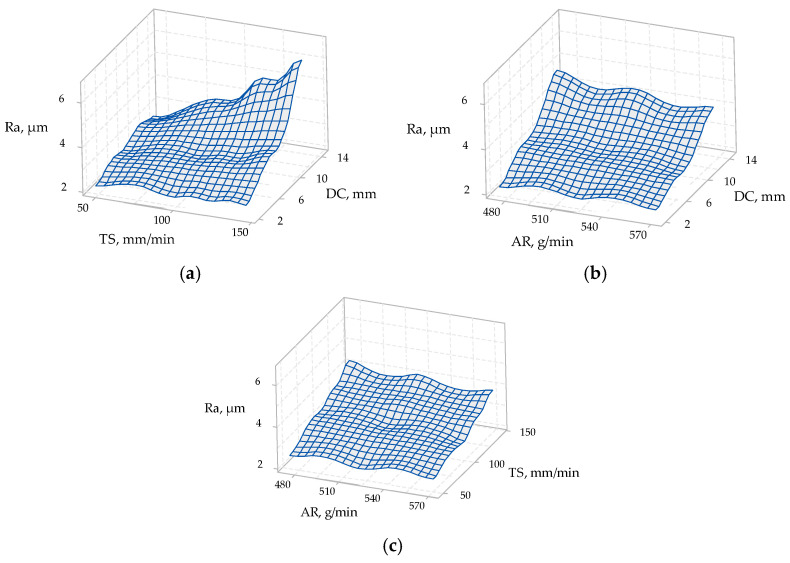
3D surface plots of process parameters vs. surface roughness of 15 mm material thickness: (**a**) traverse speed (TS) vs. depth of cut (DC) on surface roughness (*Ra*); (**b**) abrasive mass flow rate (AR) vs. depth of cut (DC) on surface roughness (*Ra*); (**c**) abrasive mass flow rate (AR) vs. traverse speed (TS) on surface roughness (*Ra*).

In the AWJ cutting of the 15 mm thick material it was also observed (see Figure 9a,b) that the depth of cut had a substantial effect on the surface roughness. The same conclusion can be drawn from Figure 7 and Figure 8, during the AWJ cutting of 5 mm and 10 mm material thicknesses.

Response surfaces comply with the findings of A. Deaconescu and T. Deaconescu [22] by researching the impact of various AWJ process parameters. They analyzed similar material with higher thicknesses but without analysis of a cut surface at different depths.

### 3.3. ANN Concerning ANOVA

Since the AR process parameter has been recognized as the least influential factor by ANOVA, it was excluded from the learning set. Instead of four input variables, the ANN’s inputs consisted of only three process parameters (DC, TS, and material thickness). By excluding AR, the ANN can model the data and provide results faster with higher precision; Table 10 presents the results. Compared to Table 4, most ANNs produced better test performance metrics, yielding a 5.75% lower test MAE and a 5.54% lower test RMSE. The ANNs presented in the table yielded similar train MAE and RMSE results compared to Table 4. From a computation time standpoint, the tested ANNs performed 12.18% faster than the ANNs in Table 4. Additionally, by considering the Section 3.1 findings, wherein the ANN 5 topology was recognized as best-suited, the ANN 5′s results with reduced data (with exclusion of AR) yielded similar improvements of the test MAE (test MAE=0.1779) and the RMSE (test RMSE = 0.2084).

## 4. Conclusions

A feed-forward ANNs method was used to predict the surface roughness in abrasive water jet cutting of X5CrNi18-10 (1.4301) stainless steel in different depths of cut for three different material thicknesses. This study’s novelty consisted in its introducing the ANNs method as a useful tool for predicting surface roughness along the entire depth of cut at AWJ cutting of stainless steel of different thicknesses. Its main benefit is the reduced time needed for experimenting. The experimental data obtained at different traverse speeds and abrasive mass flow rates were used to develop the feed-forward ANNs method to predict surface roughness. The predicted surface roughness values were compared with the measured values to show the efficiency of the ANNs. A single-layered ANN with five neurons (ANN 5) was recognized as the best-suited topology for the presented problem modeling. The best ANN 5 test set results for the MAE and the RMSE were 0.1779 and 0.2084, respectively. The predicted values were found to be in close agreement with the experimental. The average percentage error between the predicted surface roughness values and the experimental was 4.4973%. At a 95% confidence level, based on the percentage contribution of ANOVA, the dept of cut was the most significant factor on the surface roughness, followed by the traverse speed. By exclusion of AR, the ANNs performed faster and with increased precision.

In future research, more detailed discussions should be considered on the effects of other process parameters such as water jet pressure, the size and type of abrasive, and the type of material on surface roughness parameters, including 3D parameters.

## Figures and Tables

**Table 1:** Constant process parameters and their values used in the experiments.

Constant Parameters	Orifice Diameter	Focusing Tube Diameter	Water Jet Pressure	Abrasive Type	Abrasive Size (grit no)
Value	0.33 mm	1.016 mm	350 MPa	GMT garnet	80 mesh

**Table 2:** Input process parameters and their values used in the experiments.

Process Parameters	Traverse Speed (TS) (mm/min)	Abrasive Mass Flow Rate (AR) (g/min)
Material thickness 5 mm	139, 278, 347, 417	475, 522, 571
Material thickness 10 mm	76, 152, 190, 228	475, 522, 571
Material thickness 15 mm	48, 96, 120, 144	475, 522, 571

**Table 3:** *Ra* values in the machining of the workpiece with 5 mm thickness.

AR (g/min)	TS (mm/min)	Position throughout the Depth of the Cut (DC)		*Ra* (µm)				
		(mm)	Section	P1	P2	P3	P4	Mean
475	139	2	top	2.74	2.24	2.9	2.66	2.635
		3	middle	3.28	2.9	3.46	2.57	3.053
		4	bottom	3.36	3.02	3.11	3.14	3.158
	278	2	top	3.02	2.91	3.06	2.87	2.965
		3	middle	3.48	3.94	3.53	3.89	3.710
		4	bottom	4.87	3.71	4.72	3.86	4.290
	347	2	top	2.81	2.6	2.61	3.18	2.800
		3	middle	3.79	3.64	3.91	4.3	3.910
		4	bottom	4.58	4.34	4.95	4.65	4.630
	417	2	top	2.95	3.23	2.67	3.29	3.035
		3	middle	4.05	4.65	3.94	3.98	4.155
		4	bottom	4.74	5.39	5.09	4.71	4.983
522	139	2	top	2.7	3.21	2.67	2.79	2.843
		3	middle	2.86	2.91	2.89	2.92	2.895
		4	bottom	2.8	2.59	3.09	3.16	2.910
	278	2	top	3.24	2.65	3.21	2.74	2.960
		3	middle	3.92	4.01	4.51	3.67	4.028
		4	bottom	4.2	4.33	4.59	4.26	4.345
	347	2	top	3.07	3.05	3.31	3.15	3.145
		3	middle	3.82	4.05	4.32	4.25	4.110
		4	bottom	4.2	4.3	4.45	4.55	4.375
	417	2	top	3.41	3.04	3.11	3.44	3.250
		3	middle	3.85	4.34	3.88	3.8	3.968
		4	bottom	4.37	4.68	4.48	5.07	4.650
571	139	2	top	3.37	2.64	3.19	2.29	2.873
		3	middle	2.67	2.93	3.01	3.33	2.985
		4	bottom	3.1	3.15	3.04	3.01	3.075
	278	2	top	2.97	3.61	2.82	2.85	3.063
		3	middle	3.53	3.63	4.14	3.97	3.818
		4	bottom	4.01	4.33	3.72	4.33	4.098
	347	2	top	2.54	2.92	3.25	3.07	2.945
		3	middle	3.54	4.25	4.09	4.32	4.050
		4	bottom	4.26	4.17	5.07	4.32	4.455
	417	2	top	3.01	2.94	2.99	2.98	2.980
		3	middle	4.08	4.08	4.01	4.62	4.198
		4	bottom	4.31	4.89	4.43	4.66	4.573

**Table 4:** *Ra* values in the machining of the workpiece with 10 mm thickness.

AR (g/min)	TS (mm/min)	Position throughout the Depth of the Cut (DC)		*Ra* (µm)				
		(mm)	Section	P1	P2	P3	P4	Mean
475	76	2	top	2.91	2.09	2.4	2.44	2.460
		5	middle	3.08	2.72	2.9	3.34	3.010
		8	bottom	4.57	2.85	2.92	3.29	3.408
	152	2	top	2.54	2.28	2.49	2.59	2.475
		5	middle	2.63	3.35	3.66	2.85	3.123
		8	bottom	4.35	3.83	4.22	3.91	4.078
	190	2	top	2.37	2.3	2.39	2.32	2.345
		5	middle	3.85	4.15	3.39	3.69	3.770
		8	bottom	4.93	5.1	4.69	4.88	4.900
	228	2	top	2.45	2.74	2.7	2.99	2.720
		5	middle	5.15	4.87	4.88	4.6	4.875
		8	bottom	6.84	6.79	6.35	6.3	6.570
522	76	2	top	2.43	2.87	2.26	2.7	2.565
		5	middle	2.86	2.71	3.1	2.95	2.905
		8	bottom	2.91	2.89	2.96	2.94	2.925
	152	2	top	2.44	2.56	2.4	2.52	2.480
		5	middle	3.41	3.22	3.47	3.28	3.345
		8	bottom	4.28	4.14	4.8	4.66	4.470
	190	2	top	2.47	2.44	2.25	2.28	2.360
		5	middle	3.45	3.39	3.71	3.65	3.550
		8	bottom	4.83	5.01	4.69	4.87	4.850
	228	2	top	2.34	2.34	2.6	2.6	2.470
		5	middle	4.15	3.91	4.34	4.1	4.125
		8	bottom	6.58	7.03	5.95	6.4	6.490
571	76	2	top	2.69	2.44	2.81	2.49	2.608
		5	middle	3.76	3.03	3.01	2.54	3.085
		8	bottom	3.32	3.1	2.88	3.55	3.213
	152	2	top	2.23	2.55	2.96	2.38	2.530
		5	middle	3.1	3.12	3.25	3.27	3.185
		8	bottom	3.86	4.51	3.9	4.55	4.205
	190	2	top	2.17	3.2	2.86	2.56	2.698
		5	middle	2.63	3.24	4.26	3.17	3.325
		8	bottom	4.79	4.36	5.42	4.99	4.890
	228	2	top	2.47	2.7	2.83	2.3	2.575
		5	middle	3.63	3.24	4.22	3.75	3.710
		8	bottom	6.47	6.26	6.31	6.1	6.285

**Table 5:** *Ra* values in the machining of the workpiece with 15 mm thickness.

AR (g/min)	TS (mm/min)	Position throughout the Depth of the Cut (DC)		*Ra* (µm)				
		(mm)	Section	P1	P2	P3	P4	Mean
475	48	2	top	2.14	2.22	2.13	2.21	2.175
		7	middle	2.63	2.48	2.78	2.63	2.630
		13	bottom	2.28	2.99	2.57	3.28	2.780
	96	2	top	2.21	2.04	2.18	2.01	2.110
		7	middle	2.99	2.78	3.22	3.01	3.000
		13	bottom	4.43	4.57	4.39	4.53	4.480
	120	2	top	2.46	2.37	2.2	2.11	2.285
		7	middle	3.05	3.33	2.99	3.27	3.160
		13	bottom	5.02	5.34	5.46	5.78	5.400
	144	2	top	2.59	2.3	2.54	2.25	2.420
		7	middle	3.39	3.08	4.08	3.77	3.580
		13	bottom	6.42	6.79	6.55	6.92	6.670
522	48	2	top	2.15	1.95	2.31	2.11	2.130
		7	middle	2.45	2.51	2.56	2.62	2.535
		13	bottom	2.81	2.43	2.92	2.54	2.675
	96	2	top	2.17	2.31	2.26	2.4	2.285
		7	middle	2.58	2.62	3.13	3.17	2.875
		13	bottom	3.75	3.86	3.92	4.03	3.890
	120	2	top	2.61	2.32	2.54	2.25	2.430
		7	middle	3.01	3.06	3.11	3.22	3.100
		13	bottom	5.4	5.24	5.35	5.19	5.295
	144	2	top	2.22	2.25	2.36	2.39	2.305
		7	middle	3.8	3.34	3.7	3.24	3.520
		13	bottom	6.34	6.88	5.92	6.46	6.400
571	48	2	top	2.24	2.09	2.48	2.33	2.285
		7	middle	2.46	2.51	2.52	2.57	2.515
		13	bottom	2.69	2.76	2.6	2.67	2.680
	96	2	top	2.3	2.2	2.35	2.25	2.275
		7	middle	2.76	2.7	2.6	2.54	2.650
		13	bottom	3.47	3.42	3.63	3.58	3.525
	120	2	top	2.2	2.26	2.26	2.32	2.260
		7	middle	2.61	2.93	2.54	2.86	2.735
		13	bottom	5.01	4.74	5.81	4.64	5.050
	144	2	top	2.36	2.35	2.75	2.42	2.470
		7	middle	3.27	3.24	3.43	3.4	3.335
		13	bottom	5.78	6.05	5.74	5.99	5.890

**Table 6:** Tested feed-forward ANN configurations.

ANN Information	Config 1	Config 2	Config 3	Config 4	Config 5
Training procedure	Trainscg	Trainscg	Traingda	Trainlm	Trainbr
Learning epochs	200	200	200	9	14
Transfer function	logsig	purelin	logsig	logsig	logsig
Architecture	ANN 4_8	ANN 4_8	ANN 4_8	ANN 4_8	ANN 4_8
Cross validation	36-fold	36-fold	36-fold	36-fold	36-fold
Test MAE	0.2046	0.4399	0.6057	0.2525	0.2093
Train MAE	0.1390	0.4251	0.5388	0.1709	0.1715
Test RMSE	0.2397	0.5197	0.6956	0.2955	0.2477
Train RMSE	0.1772	0.5699	0.6980	0.2217	0.2209
Time (s)	118.7	124.6	101.4	60.4	68.5

**Table 7:** ANOVA results for the 5 mm material thickness.

Source	*DF*	Adj *SS*	Adj *MS*	*F*-Value	*p*-Value	*PC* (%)
AR	2	0.0056	0.00281	0.03	0.966	0.03
TS	3	5.7789	1.92618	23.72	0.000	34.83
DC	2	8.5327	4.26635	52.53	0.000	51.43
Error	28	2.2742	0.08122			13.71
Total	35	16.5911				

**Table 8:** ANOVA results for the 10 mm material thickness.

Source	*DF*	Adj *SS*	Adj *MS*	*F*-Value	*p*-Value	*PC* (%)
AR	2	0.0978	0.0489	0.14	0.866	0.2
TS	3	11.0971	3.6990	10.97	0.000	22.70
DC	2	28.2529	14.1265	41.88	0.000	57.79
Error	28	9.4451	0.3373			19.31
Total	35	48.8930				

**Table 9:** ANOVA results for the 15 mm material thickness.

Source	*DF*	Adj *SS*	Adj *MS*	*F*-Value	*p*-Value	*PC* (%)
AR	2	0.3838	0.1919	0.46	0.639	0.67
TS	3	12.3679	4.1226	9.78	0.000	21.60
DC	2	32.7138	16.3569	38.80	0.000	57.12
Error	28	11.8049	0.4216			20.61
Total	35	57.2704				

**Table 10:** Tested feed-forward ANN configurations, with the AR parameter excluded.

ANN Information	Config 1	Config 2	Config 3	Config 4	config 5
Training procedure	Trainscg	Trainscg	Traingda	Trainlm	Trainbr
Learning epochs	200	200	200	9	14
Transfer function	logsig	purelin	logsig	logsig	logsig
Architecture	ANN 4_8	ANN 4_8	ANN 4_8	ANN 4_8	ANN 4_8
Cross validation	36-fold	36-fold	36-fold	36-fold	36-fold
Test MAE	0.1939	0.4366	0.5195	0.2280	0.2117
Train MAE	0.1468	0.4233	0.4652	0.1848	0.1828
Test RMSE	0.2282	0.5166	0.6045	0.2660	0.2496
Train RMSE	0.1946	0.5724	0.6042	0.2440	0.2356
Time (s)	107.1	115.3	87.4	51.0	58.7

## Data Availability

The data that support the findings of this study are available from the corresponding author, upon reasonable request.
